# Indents

**Published:** 1908-10-15

**Authors:** 


### INDENTS.

☞ **Brief Articles of Technical or Scientific Value will receive notice and comment. Contributions invited.**

**Careless Use of Bands and Ligatures in Regulating.** Many times lasting injury is done to the gum tissue from clamp bands that slip too far on the tooth, or which are not festooned at the cervical margins to prevent cutting into the gums. The foundation for future cases of pyorrhea may be laid in the use of ligatures that bury themselves in the gum entirely down to the alveolar process. Neither of these things is necessary, but may be avoided by careful and proper handling.—W. J. BRADY, *Western Dental Journal.*

**A Method of Removing a Bridge.** When necessary to remove a bridge it may be done by the following method without mutilating the abutments or crowns: Take a piece of copper wire, 36-gauge, and 15 to 18 inches long; pass one end through the interspace near the crown to be loosened, bend the end down and around the wire several times, forming a loup. Form a loop in the other end, through which pass an instrument, and, drawing the wire taut, strike it several sharp blows with a mallet. This will jar the cement loose from the tooth. Do this at each attachment.—Dr. T. T. Baker, *Practical Points.—Brief.*

**The Use of Porcelain Mixed With Alcohol.** It is probable that any porcelain that is to be applied to an invested matrix should be mixed with alcohol. It should be mixed to a cream-like consistency and flowed into position with the point of the carver or a small brush; it is not to be carved as the porcelain that is mixed with water.

After moistening the matrix with alcohol, fill it almost to the margin with the paste that is to form the foundation of the inlay. The alcohol is then evaporated and the porcelain is fired to a high biscuit. After this firing the investment should be chilled by dipping the base of the cup in water, using care to prevent the investment from becoming moist. In case more foundation is required, a second application is made and biscuited.—Dr. J. Q. Byram, in *Items of Interest.*

**To Prevent Cement, Gutta-Percha and Temporary Stopping from Sticking to Instruments.** Take an ordinary small shallow cold-cream jar with a metal cover which screws on. Cut into the metal cover a hole of the size of a dime, and pack through it cotton saturated with olive oil or Black's 1-2-3 or any oil, making a sort of pincushion. Place over the jar a piece of chamois skin and bind it around the edge of the cover with dental floss. A tough or slight rub of the instrument on the chamois will take up enough of the oil to prevent the filling material from sticking to the instrument, and yet not enough to mix with and contaminate the material.—Dr. McCracken, *Dental Review.*

